# Altered Transfer of Momentary Mental States (ATOMS) as the Basic Unit of Psychosis Liability in Interaction with Environment and Emotions

**DOI:** 10.1371/journal.pone.0054653

**Published:** 2013-02-15

**Authors:** Johanna T. W. Wigman, Dina Collip, Marieke Wichers, Philippe Delespaul, Catherine Derom, Evert Thiery, Wilma A. M. Vollebergh, Tineke Lataster, Nele Jacobs, Inez Myin-Germeys, Jim van Os

**Affiliations:** 1 Department of Psychiatry and Psychology, School of Mental Health and Neuroscience, Maastricht University Medical Centre, Maastricht, The Netherlands; 2 Department of Interdisciplinary Social Science, University of Utrecht, Utrecht, The Netherlands; 3 Department of Human Genetics, University Hospital Gasthuisberg, Catholic University Leuven, Leuven, Belgium; 4 Association for Scientific Research in Multiple Births, Ghent, Belgium; 5 Faculty of Psychology, Open University of The Netherlands, Heerlen, The Netherlands; 6 Department of Psychosis Studies, King's College London, King's Health Partners, Institute of Psychiatry, London, United Kingdom; The University of Queensland, Australia

## Abstract

Psychotic disorders are thought to represent altered neural function. However, research has failed to map diagnostic categories to alterations in neural networks. It is proposed that the basic unit of psychotic psychopathology is the moment-to-moment expression of subtle anomalous experiences of subclinical psychosis, and particularly its tendency to persist from moment-to-moment in daily life, under the influence of familial, environmental, emotional and cognitive factors.

In a general population twin sample (n = 579) and in a study of patients with psychotic disorder (n = 57), their non-psychotic siblings (n = 59) and unrelated controls (n = 75), the experience sampling paradigm (ESM; repetitive, random sampling of momentary mental states and context) was applied. We analysed, in a within-person prospective design, (i) transfer of momentary anomalous experience at time point (*t–1*) to time point (*t*) in daily life, and (ii) moderating effects of negative affect, positive affect, daily stressors, IQ and childhood trauma. Additionally, (iii) familial associations between persistence of momentary anomalous experience and psychotic symptomatology were investigated. Higher level of schizotypy in the twins (but not higher level of psychotic symptoms in patients) predicted more persistence of momentary anomalous experience in daily life, both within subjects and across relatives. Persistence of momentary anomalous experience was highest in patients, intermediate in their siblings and lowest in controls. In both studies, persistence of momentary anomalous experience was moderated by higher levels of negative affect, daily stressors and childhood trauma (only in twins), and by lower levels of positive affect. The study of alterations in the moment-to-moment transfer of subtle anomalous experience of psychosis, resulting in their persistence, helps to explain *why* psychotic and emotional dysregulation tend to cluster in a single phenotype such as schizophrenia, and *how* familial and environmental risks increase the risk of expression of psychosis from, first, subtle momentary anomalous experience to, second, observable clinical symptoms.

## Introduction

### The basic unit of psychopathology: from diagnostic categories to reactive mental states

Although it is widely believed that mental disorders have their origin in altered cerebral function, the widely criticized [1], [2] disease categories as defined in the Diagnostic and Statistical Manual of Mental Disorders (DSM) and the International Classification of Diseases (ICD) do not map on what the brain actually does: mediating the continuous flow of meaningful perceptions of the social environment that guide adaptive behaviour. The use of ex-cathedra static diagnostic categories appears distal from neural networks that mediate dynamic adaptation to social context, which is one of the reasons why diagnostic categories as the basic unit for research may hamper scientific progress [1], [2].

Given that most mental disorders as defined in DSM and ICD represent quantitative deviation from health [1], [2], it may be argued that there is a need to re-express the basic unit of psychopathology in similar terms of quantitative variation rather than qualitative distinctions. In addition, a reformulation of the basic psychopathological unit towards dynamic reactivity, modelled on the role of neural networks in mediating adaptive functioning to social context, may be more productive. A new basic unit of psychopathology thus should include (i) quantitative variation from health to disease and (ii) dynamic daily life reactivity. Phenotypes combining dimensional variation and daily life reactivity are commonly assessed in medicine; examples are the longitudinal monitoring of blood pressure, muscle tone and brain waves in the flow of daily life. As these phenotypes are informative with regard to diagnosis, treatment needs and prognosis, introduction of similar dimensional reactive phenotypes in psychiatry may be fruitful. However, assessing constantly changing mental representations that are not directly observable in the flow of daily life requires novel, longitudinal, within-person tracking technologies that have been introduced only relatively recently.

### Prospective within-person assessment of reactive mental states in daily life: The Experience Sampling Method (ESM)

Recent work has demonstrated that ESM represents a suitable technology for within-person momentary assessments of variability and context sensitivity of evolving mental states [3]. ESM can now be carried out with an electronic device [4], [5] that signals the person at random moments during the day to input data on mental states (e.g. negative affect, positive affect, or anomalous experience indicating subthreshold psychosis) and context (e.g. minor stressful events, activity, company). Individuals typically sample experiences for a period of five or six days. ESM thus allows for the prospective within-person assessment of reactivity and context sensitivity of evolving mental states in the flow of daily life. An important prospective within-person variable in this respect is the level of transfer of a mental state from one moment to the next [6]. For example, increased transfer of momentary mental states indexing anomalous experiences [7] may represent the first phase of development of (subclinical) psychotic symptoms [8]. Examples of such mental states may be [8], [9]: peculiar experience of personal significance of certain events, heightened perception, sense of threat, actions experienced as unintended, racing thoughts, thoughts appearing to be broadcasted, thoughts experienced as voices, two unconnected events appearing to be causally linked. It has been proposed that the mechanism underlying the development of anomalous experiences represents the process of “aberrant salience” [9]: under the influence of dysregulated dopamine release, aberrant assignment of salience to otherwise neutral stimuli may result in a profound experience of personal significance, which in turn may give rise to secondary psychotic interpretations. Aberrant salience can be ascribed to multiple types of stimuli, e.g. visual, emotional or social [10]. Multiple genetic and environmental liabilities [11], [12] predispose an individual who is liable to psychosis to a dysregulated dopamine system, that releases dopamine independently (i.e., independent of cue and context [12]), causing the assignment of salience to otherwise neutral or insignificant stimuli.

Thus, dopamine has been proposed to represent the ‘final common pathway’ to psychosis along its full extended phenotype (i.e. from subclinical to clinical psychotic expression) [13], linking expression of psychosis at ‘brain-level’ with ‘mind-level’^9^. The hypothesized role of aberrant salience as the driving force behind psychotic symptoms has been supported by empirical studies showing that psychotic symptoms were associated with aberrant salience in both patients with schizophrenia and controls with increased liability for psychosis [14] and by imaging studies showing that patients with schizophrenia show abnormal neural patterns in response to neutral stimuli [15], [16]. Furthermore, support comes from work in psychopharmacology, suggesting that antipsychotic medication attenuates both the level of psychotic symptoms and the level of aberrant salience [12], [14].

**Figure 1:**
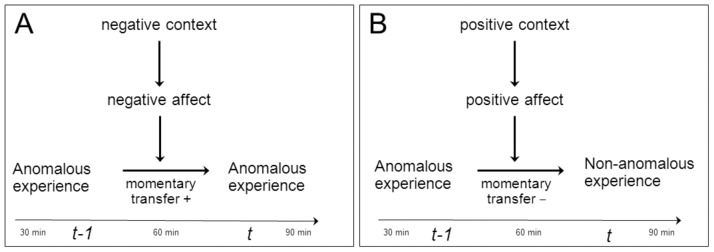
Mental state dynamics underlying formation of psychotic symptoms. In fig 1A, a momentary (note that time axis is in minutes) mental state of anomalous experience persists because the mental state is transferred from moment (*t–1*) to moment (*t*), under the influence of negative affect associated with negative environmental context. In fig. 1B, no transfer of anomalous experience occurs from (*t–1*) to (*t*), and therefore no persistence, under the influence of positive affect associated with positive environmental context. In 1A, the risk of a noticeable anomalous experience is increased whereas in 1B the risk is mitigated.

At the momentary level, an initial anomalous experience of aberrant salience (hereafter: anomalous experience) may be replaced (updated) over time by a more benign mental state, following interaction with outside context or internal representations, when the person is ‘testing’ the validity of the anomalous experience against his (internal or external) context. In the ESM series, this is represented as fluctuations in anomalous experiences from momentary time point (*t–1*) to time point (*t*). Factors such as positive affect, induced by a pleasant event or a cognitive reappraisal, may impact on the process of updating from moment to moment, giving rise to variability and context sensitivity (Fig. 1) [17]. ESM thus allows for an examination of the dynamic interaction between context, context-dependent emotional states and the level of persistence of anomalous mental states from moment to moment in daily life. It could be hypothesized that the level of persistence of momentary anomalous experience is the crucial variable determining to what degree the individual may develop observable psychotic symptoms in interaction with both proximal (e.g. environmental stress) [18] and distal (e.g. childhood trauma) [19] environmental adversity as well as with the level of familial vulnerability for expression of psychosis [20], [21]. Furthermore, given that factors such as cognitive alterations [22] and emotional context [8], [23] are strongly and intrinsically associated with expression of psychosis, it is similarly attractive to hypothesize that the origin of these intrinsic associations can be traced to their impact on the level and particularly the persistence of momentary anomalous experience.

### Advantage of within-subject designs

The use of ESM has the additional advantage that it deals with the important methodological concern [24] in the field of mental health that data are largely dependent on between-subject, cross-sectional – rather than within-subject, longitudinal – research designs. Apart from the fact that it is uncertain to what degree findings obtained by pooling across subjects can be validly applied to the individual subject [24], [25], between-subject associations are subject to confounding at a level where within-subject associations are not. For example, within the same study, the effect size of an independent variable (e.g. smoking of mother) on a dependent variable (e.g. birth weight child) may be different when measured within or between subjects. This is because the between-subject effect may additionally capture confounding by third variables, such as socio-economic status or alcohol consumption, that are absent in a within-subject design [26]. In addition, analyses using self-reports arguably make sense only in a within-subject design, since all subjects create their own starting set point. Therefore, longitudinal studies of within-subject change arguably represent the only design that allows for the examination of the true dynamics of mental states and how these may result in observable symptoms. Furthermore, within-subject designs with repeated measurements over time will be able to better deal with issues of causality and may reveal dynamic processes of mutually impacting mental states that are obscured in cross-sectional, between-subject designs.

### Aims of the study

The following questions and hypotheses were formulated.

1. Are schizotypy and psychotic disorder associated with altered transfer of momentary mental states indexing anomalous experiences? We wished to examine (i) the expression of psychotic symptoms and (ii) the psychometric expression of vulnerability for psychotic symptoms (schizotypy) as moderators of altered transfer of momentary mental states indexing anomalous experiences (momentary paranoia, hallucinatory experiences) from one moment to the other in the flow of daily life, hypothesizing that greater level of persistence of momentary anomalous experiences was associated with greater level of expression of both schizotypy in healthy subjects and psychotic symptoms in patients with psychotic disorder.
2. Is the association between altered transfer of momentary anomalous experiences and psychosis outcomes familial? This question examined to what degree altered transfer of momentary mental states indexing anomalous experiences in one relative predicted psychosis outcomes in the other. A positive association would indicate that the two phenotypes tend to co-segregate together. In addition, it was hypothesized that transfer of momentary anomalous experiences would be greatest in the patients, intermediate in their siblings, and lowest in controls.
3. Is transfer of momentary anomalous experiences moderated by emotional and cognitive factors? It was hypothesized that ESM momentary states of negative affect (increased risk) and positive affect (decreased risk) at time point (*t*) moderate the transfer of momentary anomalous experiences from time point (*t–1*) to time point (*t*). In addition, it was hypothesized that lower IQ, reflecting the level of global neurocognitive alterations in patients, would similarly increase the risk of transfer of momentary anomalous experiences.
4. Is transfer of momentary anomalous experiences moderated by environmental factors? We examined the hypothesis that both proximal momentary stress, measured in the ESM paradigm, and distal childhood trauma would increase the transfer of momentary anomalous experiences.

**Figure 2:**
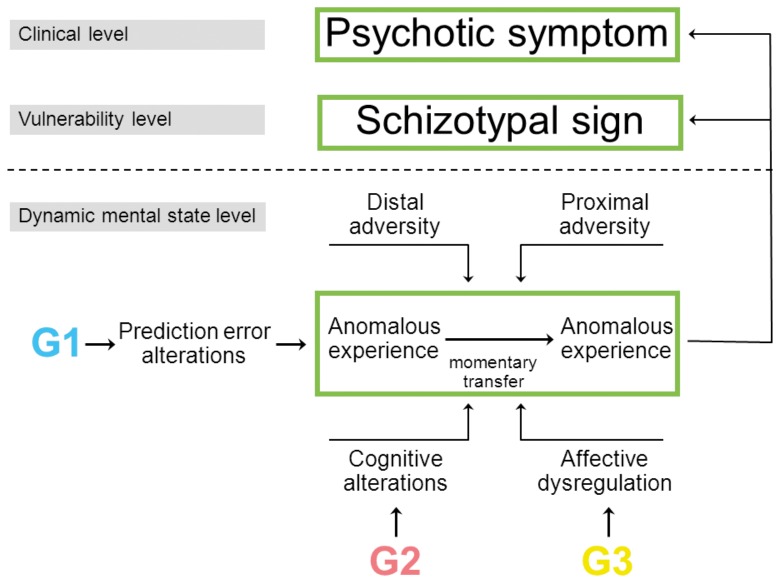
Model of psychopathology based on brain function subserving the constant updating of meaningful representations of the social environment to guide adaptive behaviour. Psychotic symptoms arise from altered transfer of momentary anomalous experiences under the influence of familial, environmental, emotional and cognitive factors. The basic unit of psychopathology underlying psychotic symptoms, situated at the level of dynamic mental states in the flow of daily life, is the probability that a mental state of momentary anomalous experience, at time point (*t–1*) will persist to time point (*t*). The probability of persistence is influenced by familial, environmental, cognitive and emotional factors, moderating the transfer of momentary anomalous experience. Higher levels of transfer of momentary anomalous experiences will result in signs and symptoms of, first schizotypy (vulnerability level) and, second, full-blown psychotic symptoms (clinical level). Familial liability may operate at several levels, including liability to experience momentary anomalous experience, and liability to experience affective and cognitive alterations impacting on transfer of momentary anomalous experience. Not included in the figure, but also relevant, is the possibility of genetic factors influencing sensitivity to environmental exposures.

The model underlying the aims and hypotheses is summarized in figure 2. The advantage of this model is that it allows for an explanation of *why* psychotic, emotional and cognitive dysregulation tend to cluster in a single phenotype, such as schizophrenia, i.e. not by assuming that all these symptoms vary together as a function of an underlying latent diagnostic construct, the evidence for which is weak [27], but because these dynamic mental states impact on each other causally [28], giving rise to observable expression of symptoms that subsequently may result in need for care. In addition, it provides an answer to the question *how* familial risk and environmental risk increase the expression of psychosis. In other words, the study of transfer of momentary anomalous experiences may shed light on the mechanism underlying phenotypic variation in psychotic disorders and on the mechanism underlying risk and resilience.

## Methods

### Study 1: General population TWIN sample (TWINS)

Data were derived from 621 female siblings (mostly twins, but also including 45 non-twin sisters), sampled from the East Flanders Prospective Twin Study register [29]. The EFPTS is a population-based register, prospectively recording all multiple births in Flanders, Belgium, since 1964. The study was approved by the ethics committee of the Maastricht University Medical Centre and all participants provided written informed consent.

The sample was assessed at five time points (T0–T4). Experience Sampling Method data (ESM, see below) were collected at baseline, as described in detail elsewhere [30], [31]. These data have been used for other studies as well, but never in the context of the current research questions [30], [32]–[42], as this is the first study analysing the novel phenotype of transfer of momentary anomalous experiences in daily life.

### Study 2: GROUP patient sample (GROUP)

The sample consisted of patients with a diagnosis of non-affective psychotic disorder, their siblings, and healthy comparison subjects from the general population in the context of the Dutch national GROUP project [43]. In selected representative geographical areas in the Southern part of the Netherlands and Belgium, patients were identified through a number of representative clinicians whose caseload was screened for inclusion criteria. Subsequently, a group of patients presenting consecutively at these services either as out-patients or in-patients were recruited for the study. Siblings were sampled through participating patients. Comparison subjects were recruited through random mailings and local paper advertisements in municipalities reflecting the mix of urbanization of the catchment areas of the mental health institutions. Inclusion criteria were (1) age range 16–50 years, (2) diagnosis of non-affective psychotic disorder, and (3) good command of the Dutch language. Diagnosis was based on DSM-IV criteria, assessed with the Comprehensive Assessment of Symptoms and History interview or Schedules for Clinical Assessment in Neuropsychiatry (version 2.1). A variable “group” was constructed, reflecting the (increasing) level of psychosis liability: 0 = controls, 1 = siblings and 2 = patients. The study was approved by the standing ethics committee of the Utrecht University Medical Centre, and all of the subjects older than 18 gave written informed consent in accordance with the committee's guidelines. For participants under the age of 18, written informed consent was provided by parents or legal guardians.

### Experience Sampling Method (ESM)

In both studies, ESM was used. ESM is a structured diary technique that addresses the daily living environment of participants [3] and possible therapeutic effects thereof [5], [44]. Participants received a wristwatch and a set of self-assessment booklets, one for each day. The wristwatch was programmed to emit a beep-signal at random moments in each of ten 90-min time blocks between 7.30 am and 13.30 pm on five (TWINS) or six (GROUP) consecutive days. The semi-random beep-design prevents anticipatory behaviour of participants. After each beep, participants were asked to complete the self-assessment booklet within 15 minutes, thus collecting reports of thoughts, current context (activity, social context, location), appraisals of the current situation and affect. In order to verify whether the subjects had completed the form within 15 min of the beep, the time at which subjects indicated they completed the report was compared to the actual time of the beep (subjects were not able to check actual beep times retrospectively). All reports completed more than 15 min after the signal were excluded from the analysis as earlier work has shown that after this interval, reports are less reliable and consequently less valid [45]. Subjects with less than 17 (TWINS) and 20 (GROUP) valid reports (out of 50) were similarly excluded [45]. Earlier work has shown that self-reported compliance, assessed in a random subset of 58 subjects (1938 observations), was very high (96.4%) [46].

### Measurements

*Affect* Adjectives of affect were rated by participants on 7-point Likert scales ranging from 1 = ”not at all” to 7 = ”very”. Following earlier work [5], [47], two factor-based scales were constructed. A “negative affect” (NA) scale was constructed based on mood adjectives such as “down”, “guilty”, “insecure”, “lonely” and “anxious” (Cronbach's alpha: 0.76 in *TWINS* and 0.82 in *GROUP*). A “positive affect” (PA) scale was constructed based on mood adjectives such as “happy”, “cheerful”, “relaxed” and “satisfied” (Cronbach's alpha: 0.86 in *TWINS* and 0.79 in *GROUP*).

#### Momentary anomalous experience In TWINS

Psychosis-like experience was based on the ESM item “I feel suspicious”, also rated on a 7-point Likert scale (1 to 7), as described in earlier work [48]. This variable is hereafter referred to as *Momentary anomalous experience*. In *GROUP*, a scale representing momentary *anomalous* experience was based on the mean score of eight ESM items on: “*I can't get rid of my thoughts*”, “*My thoughts are difficult to express*”, “*My thoughts are influenced by other people*”, “*I feel suspicious*”, “*I feel unreal*”, “*I see things that aren't really there*”, “*I hear voices*”, “*I am afraid of losing control*” (Cronbach's alpha: 0.74) in following of earlier work [49]. The use of these items indicating psychosis has been validated previously [49]–[51]. This variable is hereafter also referred to as *Momentary anomalous experience*.

#### Ratio of Momentary Anomalous experience

In both samples, the ratio of persistence to non-persistence of momentary anomalous experience was calculated as follows. First, ESM moments of momentary anomalous experience were defined by a cut-off of 2 on the Likert scale (0 = no momentary anomalous experience; 1 = yes momentary anomalous experience). Next, persistence of momentary anomalous experience was defined for each moment (0 = no persistence; 1 = yes persistence, when momentary anomalous experience, thus defined, was present at two consecutive moments).

#### Event stress

Consistent with earlier work [49], event stress was conceptualized as subjective appraised stressfulness of small daily events. After each beep, subjects rated the most important recent event on a 7-point Likert scale (−3 = very unpleasant to 3 = very pleasant). This score was used as an indicator of event-related stress in both *TWINS* and *GROUP* and is hereafter referred to as *Event stress*.

#### Early trauma In TWINS

Childhood trauma was assessed using a self-report questionnaire based on the Dutch version of the 25-item short form of the Childhood Trauma Questionnaire [52], omitting 4 of the most explicit items concerning sexual and physical abuse at the request of EFPTS. Participants rated the frequency of the (positive or negative) experience during childhood and/or adolescence on a scale from 1 (‘‘never’’) to 5 (‘‘always’’). Positive events were recoded so that higher scores reflected more adverse experiences. The continuous variable “trauma”, reflecting the weighted mean score of the 21 items of the questionnaire. In *GROUP,* childhood trauma was assessed with the Dutch version of the 25-item short form of the Childhood Trauma Questionnaire, consistent with earlier work [53]. The trauma score reflected the mean item score.

*IQ* (*GROUP only)* Overall intellectual functioning in GROUP was measured with the four-subtest version (Information, Block design, Digit Symbol Substitution Test, Arithmetic) of the Wechsler Adult Intelligence Scale-III (WAIS-III) [54].

#### (Subclinical) Positive psychotic symptoms In TWINS

the level of stable psychometric expression of psychosis was assessed with the Symptom Checklist-90-R (SCL-90-R) [55], a reliable and valid screening instrument for a range of symptoms occurring in the last week, administered at all time points. Consistent with previous work [56], [57], the SCL-90 subscales on psychoticism and paranoid ideation, which rate a broader psychosis phenotype indexing the personality – or schizotypal – dimension of psychosis, were averaged to create an *SCL-schizotypy* score at each time point. In patients in the *GROUP* sample, the level of positive symptoms occurring in the last week was assessed with the Positive and Negative Syndrome Scale (PANSS) [58]. The score of the positive symptom subscale in the patients was used as a measure of the level of positive symptoms. In the siblings of the patients, subclinical psychosis symptom dimensions were assessed with the Structured Interview for Schizotypy – Revised [59]. The SIS-R is a semi-structured interview containing 20 schizotypal symptoms (verbal responses to standardized questions) and 11 schizotypal signs (behaviours rated by the interviewer), rated on a 4-point scale. The questions and rating procedures are standardized. As described previously [43], the item scores were grouped into two dimensional scores, representing the mean scores for positive schizotypy items (referential thinking, psychotic phenomena, derealization, magical ideation, illusions, and suspiciousness) and negative-disorganized schizotypy items (social isolation, sensitivity, introversion, restricted affect, disturbances in associative and goal-directed thinking, poverty of speech, eccentric behaviour). These variables are hereafter referred to as *SIS-positive schizotypy* and *SIS-negative schizotypy*.

To facilitate the visualization of interactions, scores on NA, PA, trauma, and, when available, IQ and schizotypy, were split at the median creating a dichotomous indicator of higher or lower than inter/intra-person average levels of the respective measures. Interactions with variables measured as ESM beep-level (i.e. multiple measurements per day within each person such as NA, PA and *Event stress*) reflect intra-individual moderation; interactions modelled with person-level variables (e.g. early trauma, IQ, schizotypy) reflect inter-individual level moderation.

### Analyses

All analyses were done in Stata 12 [60]. The rate of transfer of momentary anomalous experience was tabulated in the different samples, by calculating the frequency of the ESM-measures of momentary anomalous experience, dichotomised around value 1 (absence), persisting from moment (*t–1*) to moment (*t*). In addition, specific analyses were carried out to examine each hypothesis as detailed below. All interactions were tested with continuous moderator variables, followed by stratification of effects around the median of the moderator to aid graphic visualization.

1. Are schizotypy and psychotic symptoms associated with altered transfer of momentary anomalous experience? The model of transfer of momentary anomalous experience in the ESM framework was the association between *Momentary anomalous experience at (t–1)* (independent variable) and *Momentary anomalous experience at (t)* (dependent variable). In the TWINS general population sample, we predicted that transfer of momentary anomalous experience would be stronger (i.e. the association between *Momentary anomalous experience at (t–1)* and *Momentary anomalous experience at (t)* would be stronger) in individuals with higher levels of *SCL-schizotypy*. This was tested by fitting an interaction between *Momentary anomalous experience at (t–1*) and *SCL-schizotypy*. In the GROUP patient sample, this was tested by fitting an interaction between *Momentary anomalous experience at (t–1)* and *PANSS positive symptoms.*
2. Is the association between psychotic symptoms/disorder and altered transfer of momentary anomalous experience familial? In TWINS, this was tested by examining to what degree transfer of momentary anomalous experience in the proband twin varied as a function of SCL-schizotypy in the co-twin. In GROUP, this question was tested in two ways. First, we analysed the prediction that transfer of momentary anomalous experience would be greatest in the patients, intermediate in the siblings, and lowest in the controls. Second, the level of transfer of momentary anomalous experience in patients was analysed as a function of level of *SIS-positive schizotypy/SIS-negative schizotypy* in the non-psychotic siblings.
3. Is transfer of momentary anomalous experience moderated by emotional and cognitive factors? In order to examine the impact of emotional factors (daily life NA and PA assessed in both TWINS and GROUP) and cognitive factors (IQ in GROUP) on transfer of momentary anomalous experience, the interaction between these factors and *Momentary anomalous experience at (t–1)* was examined when predicting *Momentary anomalous experience at (t)*.
4. Is transfer of momentary anomalous experience moderated by environmental factors? In order to examine the impact of early trauma (mean CTQ score in both TWINS and GROUP) and daily stress (Event stress) on transfer of momentary anomalous experience, the interaction between these factors and *Momentary anomalous experience at (t–1)* was examined when predicting *Momentary anomalous experience at (t)*.

ESM data have a hierarchical structure with multiple observations (level 1) nested within individuals (level 2) who, in turn, were nested within families (GROUP) or twin pairs (TWINS) (level 3). Given that hierarchical clustering induces violation of the assumption of independence of observations, standard errors were corrected for hierarchical clustering of observations within these levels by applying multilevel random regression models, or statistical models of parameters that vary at more than one level. Multilevel models are ideally suited for research designs where the data for participants are organized at more than one level.

The Stata XTMIXED routine was used to accommodate the three levels of hierarchical clustering, yielding non-standardized regression coefficients. Time-lagged multilevel analyses were used to examine transfer of momentary anomalous experience prospectively over time, and moderation thereof by emotional and cognitive factors. For example, the model of transfer of momentary anomalous experience, and moderation thereof by negative affect (NA) can be modelled mathematically as follows for the i-th momentary observation score of subject j:

Momentary anomalous experience (MAE) _ij t_ =  β_0_ + β_1_ MAE _ij t–1_ + β_2_ NA_ij t_ + β_3_ MAE _ij t–1_*NA_ij t_ + ζ_1j_ + ζ_2j_MAE_ij t–1_ +ε_ij_. Here, ζ_j_ represents the subject's deviation from the overall mean (random intercept) and ζ_2j+_MAP_ij t–1_ is the subject's deviation from the overall slope (random slope). The addition of ζ_2j_MAE_ij t–1_ is conservative, avoiding type I error associated with bias in mixed model inference for fixed effects [61].

## Results

### Samples

#### TWINS

Of the 621 subjects, 610 participated in the ESM study. Thirty-one participants were excluded because they had not enough (<30%) valid data points, leaving 579 individuals with data on momentary paranoia, SCL-positive schizotypy, and NA. Data on PA was available for 567 individuals. Subjects were aged between 18 and 61 years (mean age 27.7 (SD 7.9) years). The majority (64%) had a college or university degree, 34% had completed secondary education and 2% had completed primary education only. The majority was in a relationship (77%) and participants were employed (61%), studying (36%), or not working (unemployed, on sick leave or retired) (3%) at baseline. Mean level of NA was 1.27 (SD 0.37), of PA 4.43 (SD 0.86), of SCL-positive schizotypy 0.26 (SD 0.32) and of momentary anomalous experience 1.16 (SD 0.33).

#### GROUP

Fifty-seven patients participated in the ESM study, as well as 59 siblings and 75 controls. Baseline descriptives of this sample can be found in Table 1.

**Table 1:** Descriptives of GROUP patient sample with Experience Sampling measures.

	Patients	Siblings	Controls
N	57	59	75
Mean(SD) age	27.3 (8.2)	26.5 (7.6)	31.9 (10.3)
% female	35%	67%	73%
Education			
Only primary/secondary school	6%	2%	4%
Vocational education	71	43%	28%
College/University	23%	55%	68%
Mean IQ (SD)	102.7 (15.3)	108.5 (15.3)	112.0 (14.2)
ESM Mean momentary anomalous experience (SD)	1.47 (0.58)	1.13 (0.23)	1.13 (0.23)
ESM Mean NA (SD)	1.76 (0.75)	1.31 (0.53)	1.30 (0.32)
ESM Mean PA (SD)	4.44 (1.00)	5.00 (0.85)	4.97 (0.67)
ESM Mean event stress (SD)	1.62 (0.83)	1.59 (0.64)	1.52 (0.64)
Mean trauma (SD)	1.72 (0.40)	1.49 (0.31)	1.50 (0.35)

ESM  =  Experience Sampling Method.

### Prevalence of momentary anomalous experience

#### TWINS

There were 19253 observations of momentary anomalous experience in the ESM paradigm. Of these, 17687 (92.0%) were non-momentary anomalous experience, 879 (4.5%) were momentary anomalous experience that did not persist to the next moment, and 687 (3.5%) were moments of momentary anomalous experience that did persist to the next moment. Thus, the ratio of persistence to non-persistence was 0.78 (3.5/4.5).

#### GROUP

In the control group, there were 3061 ESM moments. Of these, 300 (9.8%) were aberrant momentary anomalous experience that did not persist to the next moment, and 426 (13.9%) were moments of momentary anomalous experience that did persist to the next moment. Relatives collected 2243 ESM observations, of which 240 (10.7%) were momentary anomalous experience moments that did not persist to the next moment, and 285 (12.7%) were moments of momentary anomalous experience that did persist to the next moment. Patients provided 2108 ESM observations, of which 364 (17.3%) were momentary anomalous experience moments that did not persist to the next moment, and 622 (29.5%) were moments of momentary anomalous experience that did persist to the next moment. The ratio of persistence to non-persistence in controls, relatives and patients thus was 1.4, 1.2 and 1.8 respectively.

### 1. Are schizotypy and psychotic symptoms associated with altered transfer of momentary anomalous experience?

#### TWINS

**Figure 3:**
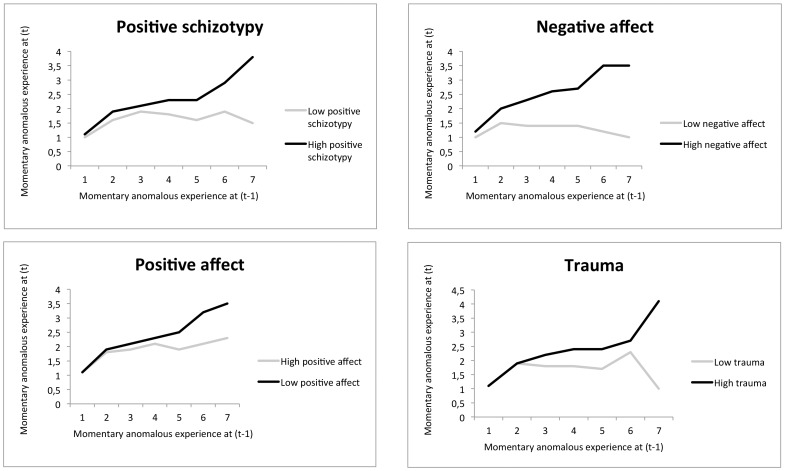
TWIN general population sample: Experience sampling measures of Momentary anomalous experience at (t–1) predicting Momentary anomalous experience at (t) for lower and higher levels (median split) of respectively positive schizotypy, negative affect, positive affect, and early trauma.

SCL-schizotypy was associated with momentary anomalous experience (B = 0.43, 95% CI 0.35, 0.51, *p*<.001). Moreover, there was a significant interaction between *SCL-schizotypy* and *Momentary anomalous experience* at (*t–1)* predicting *Momentary anomalous experience* at (*t*), with increased transfer of momentary anomalous experience being associated with higher levels of *SCL-schizotypy* (B = 0.11, 95% CI 0.05, 0.17, *p*<0.001). Thus, in individuals with low levels of *SCL-schizotypy*, transfer of momentary anomalous experience was less pronounced (B = 0.04 95% CI 0.00, 0.09, *p*<0.075) than in individuals with high levels of *SCL-schizotypy* (B = 0.13, 95% CI 0.10, 0.16, *p*<0.001) (Fig. 3).

#### GROUP

There was no significant interaction between *PANSS positive symptoms* and *Momentary anomalous experience* at (*t–1*) predicting *Momentary anomalous experience* at (*t*) (B = 0.01, 95% CI 0.00, 0.02, *p*<0.114). Thus, patients with lower levels of *PANSS positive symptoms* (B = 0.30, 95% CI 0.13, 0.47, p<0.001) did not have significantly less transfer of momentary anomalous experience than patients with higher levels of *PANSS positive symptoms* (B = 0.39, 95% CI 0.24, 0.55, *p*<0.001), although the effect was in the expected direction.

### 2. Is the association between psychotic symptoms/disorder and altered transfer of momentary anomalous experience familial?

#### TWINS

SCL-schizotypy in the co-twin was associated with momentary anomalous experience in the proband twin (B = 0.15, 95% CI 0.07, 0.23; *p*<.001). However, there was no significant interaction between *SCL-schizotypy* in the co-twin and *Momentary anomalous experience* at (*t–1*) predicting *Momentary anomalous experience* at (*t*) (B = 0.01, 95% CI −0.06, 0.08, p<0.795). Thus, individuals with higher levels of schizotypy in the co-twin (B = 0.11, 95% CI 0.07, 0.14, *p*<0.001) did not have significantly more transfer of momentary anomalous experience than individuals with lower levels of schizotypy in their co-twin (B = 0.07, 95% CI 0.02, 0.11, *p*<0.002), although the effect was in the expected direction.

#### GROUP

There was a suggestive interaction between group and *Momentary anomalous experience* at (*t-1*) predicting *Momentary anomalous experience* at (*t*) (B = 0.07, 95% CI 0.00, 0.14, *p*<0.052). Thus, in controls (B = 0.21, 95% CI 0.11, 0.32, *p*<.001), siblings (B = 0.31, 95% CI 0.19, 0.42, *p*<0.001) and patients (B = 0.35 95% CI 0.26, 0.44, *p*<0.001), *Momentary anomalous experience* at (*t-1*) predicted *Momentary anomalous experience* at (*t*) progressively more strongly, suggesting a dose-response association. There was no interaction between *SIS-positive schizotypy* (B = 0.12, 95% CI −0.02, 0.27, *p*<0.091) or SIS-negative schizotypy (B = −0.03, 95% CI −0.22, 0.16, *p*<0.755) in the sibling and patient *Momentary anomalous experience* at (*t–1*) in the model of patient *Momentary anomalous experience* at (*t*). Thus, patients with higher levels of positive schizotypy in their sibling (B = 0.44, 95% CI 0.24, 0.64, *p*<0.001) did not have significantly less transfer of anomalous experience than patients with lower levels of positive schizotypy in their sibling (B = 0.25, 95% CI 0.08, 0.43, *p*<0.001). Likewise, patients with higher levels of negative schizotypy in their sibling (B = 0.41, 95% CI 0.23, 0.59, *p*<0.001) did not have significantly more transfer of anomalous experience than patients with lower levels of negative schizotypy in their siblings (B = 0.24, 95% CI 0.04, 0.44, *p*<0.018). However, both effects were in the expected direction.

### 3. Is transfer of Momentary anomalous experience moderated by emotional and cognitive factors?

#### TWINS

There was a significant interaction between NA at (*t*) and *Momentary anomalous experience* at (*t–1*) in the model of *Momentary anomalous experience* at (*t*) (B = 0.19, 95% CI 0.17, 0.21, *p*<.001), indicating more transfer of momentary anomalous experience in individuals with higher levels of NA (B = 0.19, 95% CI 0.17, 0.22, *p*<0.001) compared to individuals with lower levels of NA (B = −0.15, 95% CI −0.18, −0.12, *p*<0.001). Similarly, there was a significant interaction between PA at (*t*) and *Momentary anomalous experience* at (*t–1*) in the model of *Momentary anomalous experience* at (*t*) (B = −0.08, 95% CI −0.10, −0.07, *p*<0.001), indicating that individuals with lower levels of PA had higher levels of transfer of momentary anomalous experience (B = 0.14, 95% CI 0.11, 0.16, *p*<0.001) compared to individuals with higher levels of PA (B = 0.03, 95% 0.00, 0.06, *p*<0.072) (Fig. 3).

#### GROUP

**Figure 4:**
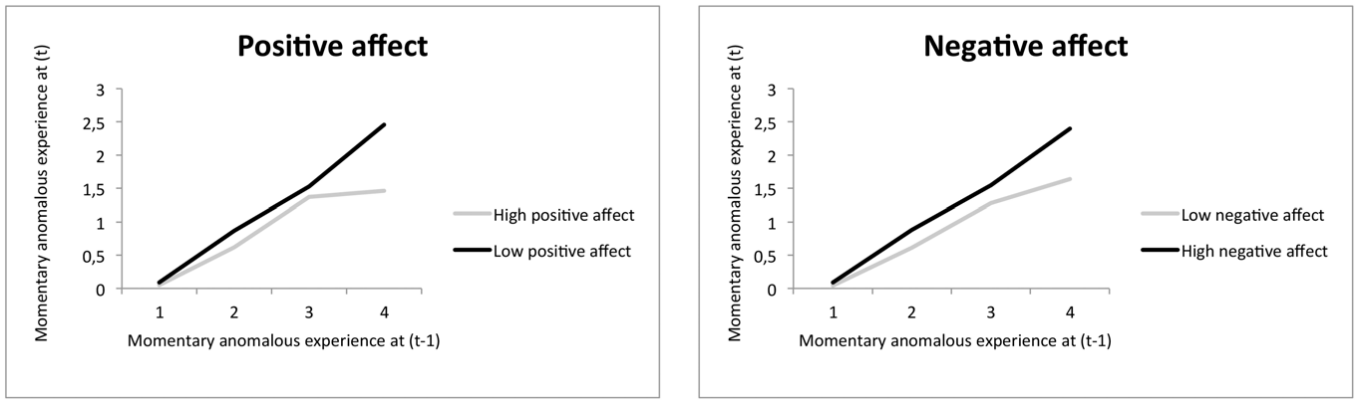
GROUP patient sample: Experience sampling measures of Momentary anomalous experience at (t-1) predicting Momentary anomalous experience at (t) for lower and higher levels (median split) of positive affect and negative affect.

Within the patient group, there were significant moderating effects of PA (B = −0.15, 95% CI −0.17, −0.12, *p*<0.001) and NA (B = 0.15, 95% CI 0.13, 0.17, *p*<0.001) on the association between *Momentary anomalous experience* at (*t-1*) and *Momentary anomalous experience* at (*t*), indicating higher level of transfer of momentary anomalous experiences with higher levels of NA, and lower levels of PA. Thus, momentary transfer of anomalous experience was stronger in patients with high NA (B = 0.42, 95% CI 0.32, 0.52, *p*<.001) than in patients with low NA (B = 0.17, 95% CI 0.07, 0.28, *p*<0.001). Likewise, momentary transfer of anomalous experience was stronger in patients with low PA (B = 0.47, 95% CI 0.37, 0.57, *p*<.001) than in patients with high PA (B = 0.24, 95% CI 0.14, 0.35, *p*<0.001). There was no such moderating effect for IQ (B = 0.00, 95% CI 0.00, 0.01, *p*<0.99). Thus, patients with lower levels of IQ (B = 0.45, 95% CI 0.27, 0.62, p<0.001) did not have significantly more transfer of anomalous experience than patients with higher levels of IQ (B = 0.31, 95% CI 0.17, 0.46, *p*<0.001), although effects were in the expected direction. Graphic representation of the significant effects around the intra-person median split of moderator variables is depicted in Fig. 4.

### 4. Is transfer of Momentary anomalous experience moderated by environmental factors?

#### TWINS

ESM *Event stress* in daily life moderated the transfer of *Momentary anomalous experience* (B = 0.06, 95% CI 0.05, 0.08, *p*<.001), increasing event stress being associated with more momentary transfer of psychosis from (*t–1*) to (*t*) in a dose-response fashion (Table 2). Trauma also moderated the transfer of *Momentary anomalous experience* (B = 0.12, 95% CI 0.07, 0.17, *p*<0.001), indicating that individuals with less trauma (B = 0.04, 95% CI 0.00, 0.07, *p*<0.056) showed less transfer of momentary anomalous experience compared to individuals with more trauma (B = 0.16, 95% CI 0.12, 0.19, *p*<0.001).

**Table 2:** TWIN general population sample and GROUP patient sample: Unstandardized coefficients (B) of ESM momentary anomalous experience at (*t–1*) predicting ESM momentary anomalous experience at (*t*) with increasing appraised ESM stressfulness of events.

	TWIN study		GROUP study	
Appraised stressfulness of daily events	B	p-value	B	p-value
Very pleasant	0.01	0.583	0.28	0.001
Pleasant	0.04	0.052	0.35	0.001
A bit pleasant	0.05	0.061	0.34	0.001
Neutral	0.14	0.001	0.46	0.001
A bit unpleasant	0.12	0.002	0.46	0.001
Unpleasant	0.31	0.001	0.04	0.660
Very unpleasant	0.46	0.001	0.62	0.001

#### GROUP

Within the patient group, there was a significant moderating effect of ESM *Event stress* (B = 0.04, 95% CI 0.02, 0.06, *p*<0.001) on the association between *Momentary anomalous experience* at (*t–1*) and *Momentary anomalous experience* at (*t),* indicating more momentary transfer with higher levels of ESM *Event stress*. Thus, more exposure to events in daily life was associated with more transfer of *Momentary anomalous experience* from (*t-1*) to (*t*) (Table 2). Early trauma did not have such a moderating effect (B = 0.01, 95%CI –0.24, 0.25, *p*<0.953). Thus, patients with more trauma (B = 0.35, 95% CI 0.20, 0.50, p<0.001) did not have significantly more transfer of momentary anomalous experience than patients with less trauma (B = 0.36, 95% CI 0.20, 0.53, *p*<0.001).

## Discussion

Higher level of schizotypy in well twins (but not higher level of psychotic symptoms in patients – although directionally similar) prospectively predicted greater probability of persistence of momentary anomalous experiences over time in daily life. Although not moderated by present level of psychotic symptoms, persistence of momentary anomalous experience was greatest in the patients, intermediate in their siblings and lowest in controls. Altered transfer of momentary anomalous experience was not familial in the healthy twins, but in both healthy twins and patients with psychotic disorder, altered transfer of momentary anomalous experience from time point (*t–1*) to time point (*t*) in the daily life ESM paradigm was predicted by higher levels of negative affect, lower levels of positive affect and higher levels of minor stressors at (*t–1*). In addition, altered transfer of momentary anomalous experience was predicted by more exposure to childhood trauma in twins. Although extensive research has demonstrated a link between cognitive, environmental and familial factors on the one hand, and expression of psychosis on the other, this paper is the first, to our knowledge, to show the importance of such factors in the *persistence* of anomalous experiences of subclinical psychosis from moment to moment in daily life in individuals with vulnerability for psychosis and to suggest that altered transfer of momentary mental states (ATOMS) may be the basic unit underlying liability for psychosis, in interaction with an individual's internal and external context.

### Altered transfer of momentary anomalous experience as the basic unit of psychosis

The hypothesis that altered transfer of momentary anomalous experience may represent the first phase of development of psychotic symptoms was examined using ESM methodology. Whereas psychosis items in the TWIN sample were limited to a single item of paranoia, ESM items were more elaborate in the GROUP sample. The items in this dimension capture a broader range of experiences (e.g. feeling of losing control, hearing voices); however, these items have been shown earlier to reflect the construct of psychosis [42]–[44]. These ESM items in GROUP yielded a more sensitive phenotype, as indicated by higher rates of momentary anomalous experiences, dichotomously defined, in the GROUP control sample (24%) than in the TWIN sample (8%).

Within-person prospective modelling of changes in mental states associated with subthreshold psychosis indicated that greater moment-to-moment persistence of momentary anomalous experience was associated with higher levels of schizotypy in well twins, although it was not associated with higher levels of psychotic symptoms in patients. These results suggest that psychosis, in the phase of subclinical expression and suggestively along the full spectrum of the extended psychosis phenotype, can be represented at the level of altered transfer of momentary mental states (ATOMS) in the flow of daily life.

### Elucidating the mechanism of phenotypic heterogeneity: Momentary interactions underlying symptoms

Research suggests that patients meeting diagnostic criteria for schizophrenia in practice differ from each other more than they have in common with regard to symptoms, course and aetiology [62]. Symptom dimensions of affective, psychotic, negative and cognitive symptoms are loosely correlated with each other at the level of the general population [63]; this explains their tendency to co-occur to a degree in the same syndrome. However, the marked heterogeneity in psychopathology suggests that symptoms also causally impact on each other over time, contributing to the markedly different combinations in different patients observed in clinical practice [28], [64]–[66]. The results of the current investigation demonstrate that the development of psychosis is a dynamic interactive process that occurs when neurocognitive variables, reflecting neurodevelopmental impairment, and emotional variables, reflecting affective dysregulation (negative affect) or resilience (positive affect), moderate the probability of persistence of momentary anomalous experience in the flow of daily life, to the degree that observable psychotic symptoms may ensue. The fact that no moderation by neurocognitive variables was found in the patients may be explained by the fact that the measure used (IQ) was too broad to capture moderation of altered transfer of momentary anomalous experience.

### Elucidating aetiological mechanisms at the level of experience

As moment-to-moment persistence of momentary anomalous experience was additionally sensitive to familial effects (in GROUP) as well as to effects of both distal (in TWINS) and momentary proximal environment (in both studies), the findings indicate that a fine-grained model of psychopathology may be productive in elucidating the role of genes and environment in the development of mental disorders such as psychotic illness. Furthermore, these complementing but only partly overlapping results indicate that at different stages of the extended psychosis continuum, different factors may play different roles. The within-person paradigm of ESM increases power, given that one individual yields multiple observations, and allows for modelling of momentary (micro)environmental and mental state variables that cannot be observed at the level of diagnostic categories or traditional symptom measures [3]. Thus, the model of altered transfer of momentary anomalous experience may provide a direct way to study aetiological mechanisms at the level of experience.

### Strengths and limitations

An important strength of the study is the replication of (directionally similar or significant) results across genetically sensitive patient and population samples, using identical momentary assessment technology. Nevertheless, findings may not be generalizable to all clinical and non-clinical populations, given that (i) twins may differ in crucial aspects from the general population and only female twins of relative high educational level were included; and (ii) the results of the clinical sample of the GROUP study may be specific for patients with recent onset psychotic disorder. Another limitation is that participants had to comply with a paper-and-pencil diary protocol. Some authors questioned the reliability and subject compliance in paper-and-pencil ESM studies, favouring the use of electronic devices. In a comparative study, Green and colleagues [67], however, concluded that both methods yielded similar results. A study by our group also showed the validity of the paper-and-pencil data [46] and in a pilot-study, we observed that backfilling (per day or week) did take more time and, moreover, was experienced as being more stressful than performing the study according to the standard protocol. In the current study, participant's compliance with the ESM protocol was checked several times. Furthermore, participants reported the time after answering the questions and only reports were included that occurred within a 20-minutes time window. Some of the interactions that were modelled consisted of variables that were not independent, thus making it difficult to distinguish between moderation or mediation. For example, a positive interaction between NA at moment (*t*) and Momentary anomalous experience at *(t–1)* in the model of Momentary anomalous experience at *(t)* may indicate that NA at moment (*t*) moderates the impact of momentary anomalous experience at (*t–1*) on momentary anomalous experience at moment (*t*), but alternatively may indicate that momentary anomalous experience at moment (*t*–1) increases NA at moment (*t*) which in turn predicts anomalous experience at moment (*t*). Both interpretations are of importance, but cannot be distinguished easily. Future studies collecting more extended ESM series may differentiate between mediation and moderation using more elaborate lagged modelling over time.
